# Exploring Psoriasis Inflammatory Microenvironment by NanoString Technologies

**DOI:** 10.3390/jcm12216820

**Published:** 2023-10-28

**Authors:** Alessia Andrea Ricci, Paolo Dapavo, Luca Mastorino, Gabriele Roccuzzo, Samanta Wolff, Simone Ribero, Paola Cassoni, Rebecca Senetta, Pietro Quaglino

**Affiliations:** 1 Pathology Unit, Department of Medical Sciences, University of Turin, 10126 Turin, Italy; alessiaandrea.ricci@unito.it (A.A.R.); paola.cassoni@unito.it (P.C.); 2 Department of Medical Sciences, Section of Dermatology, University of Turin, 10126 Turin, Italy; paolo.dapavo@unito.it (P.D.); luca.mastorino@unito.it (L.M.); gabriele.roccuzzo@unito.it (G.R.); samanta.wolff@unito.it (S.W.); pietro.quaglino@unito.it (P.Q.); 3 Pathology Unit, Department of Oncology, University of Turin, 10126 Turin, Italy; rebecca.senetta@unito.it

**Keywords:** psoriasis, immune profile, dimethyl fumarate, NanoString

## Abstract

Psoriasis is a chronic inflammatory skin disease whose molecular mechanisms and microenvironment are poorly understood. We performed gene expression analysis through the nCounter^®^ PanCancer Immune Profiling Panel (NanoString Technologies, Seattle, WA, USA) on 22 FFPE punch biopsies from 19 psoriasis-affected patients. A subset of five cases was analyzed before (T0) and after 6 months (T6) of treatment with dimethyl fumarate (DMF) to address immune microenvironment changes. Molecular comparisons according to biopsy site and age of onset showed a different distribution of innate immune cells (mast cells, macrophages, NK cells, and DC cells) and pathways (complement regulation and transporter functions). The analysis according to PASI (Psoriasis Area and Severity Index) led to non-significant results, suggesting no link between molecular expression profile and clinical amount of skin disease. In DMF-treated patients, we observed a strong immunomodulatory effect after treatment: A subversion of exhausted CD8 T cells, NK CD56dim cells, Tregs, neutrophils, CD45+ cells, T cells, B cells, and macrophages was reported between the two analyzed time-points, as well as the reduction in pro-inflammatory pathways and molecules, including cytotoxicity, pathogen defense, antigen processing, adhesion, cell cycle, chemokines, cytokines, and interleukins. The inflammatory psoriatic microenvironment can be modulated using DMF with encouraging results, achieving an immune-tolerant and non-inflammatory condition through the regulation of both innate and adaptive immunity.

## 1. Introduction

Psoriasis is a frequent chronic immune-mediated inflammatory skin disease influenced by genetic, environmental, and immunological factors. The estimated prevalence in Western countries is 2–4%, with a dual peak between 30–39 years and 60 years [1,2]. Treatment is chosen according to disease severity as assessed by the Psoriasis Area and Severity Index (PASI), ranging from 0 to 72, and considering clinical parameters of lesions [1]. Psoriasis can manifest with several distinct clinical phenotypes, mainly distinguished as plaque-type, guttate, pustular, and erythroderma psoriasis [3]: The first one is the most common, with an incidence near 90%, and can be further distinguished as Type I psoriasis (early onset <40 years) and Type II psoriasis (late onset >40 years) [4]. The other subtypes are less common, with erythroderma psoriasis being the rarest form with the most severe phenotype, observed only in 1–2% of patients [1]. From a histological point of view, several alterations can be observed, including thickening of the epidermis (a process named acanthosis), a thinned or absent granular layer, elongated and dilated capillaries, suprapapillary thinning, and an inflammatory infiltrate of T cells in the dermis and epidermis. Sometimes, clusters of neutrophils in the parakeratotic scale can be present, forming pustules (Kogoj spongiform micropustules) or be surrounded by parakeratosis (microabscesses of Munro), constituting pathognomonic signs of psoriasis [5]. Whatever its subtype, psoriasis has been demonstrated to develop in genetically predisposed individuals (heritability is the main risk determinant for developing psoriasis [6]) in which a dysregulated immune response occurs, following exposure to specific environmental triggers, such as stress, infections, trauma, alcohol consumption, smoking, and exposure to sunlight or drugs (e.g., lithium, antimalarials, non-steroidal inflammatory agents) [7,8,9,10,11]. Ultimately, its pathogenesis implies a dysregulated cross-talk between epithelial and immune cells driven by the well-established proinflammatory molecules tumor necrosis factor (TNF), interleukin (IL)-23, and IL-17, along with other mediators such as interferon (IFN)-α, IFN-γ, and IL-22, which are involved in different phases of the disease. IL-23 plays an important role in the maintenance and expansion of IL-17-producing cells [12], and it is closely related to IL-12, which can promote Th1 production of IFN-γ. Despite the fact that IL-12 and IL-23 share the p40 subunit, a therapeutic target, the direct inhibition of IL-12 is ineffective [13]. On the other hand, IL-17 is a family of six structurally related cytokines strongly involved in psoriasis pathogenesis and mainly derived from activated T cells. IL-17 leads to the upregulation of a Th17 chemoattractant, CCL20, and causes a positive IL-17 response feedback loop [14]. In the psoriatic plaque, T cells are also a source of IFN-γ, which promotes antigen processing and expression of MHC class II molecules on antigen-presenting cells, as well as of proinflammatory mediators like CXCL9 and CXCL10 that attract additional Th1 and cytotoxic T cells type 1 to the site of inflammation, setting up an additional positive feedback loop [15,16]. Lately, another interleukin family gained interest in psoriasis development, IL-36: Its expression can be induced by some of the aforementioned inflammatory mediators and can contribute to the “feed-forward amplification in psoriasis” by promoting progressively increased inflammatory activity and recruitment of other immune cells, particularly neutrophils [17]. Some of the clinical signs and symptoms of psoriasis may be explained by the equilibrium between these cytokine circuits, which contribute to psoriasis subtype manifestation. For instance, IL-23 and IL-17 prevail in plaque psoriasis [18], interferon responses are most prominent in early, developing plaques [19], while the IL-36 circuit tends to be hyperactive in pustular forms [20]. There is a mutual and self-sustaining amplification between these inflammatory mediators, with IFN-γ driving IL-23 and Th17 responses, which in turn promote IL-36 expression [21]. Since these processes are reflected in both adaptive and innate immunity regulation, the balance of CD8+ T lymphocytes and Tregs, as well as the activity of dendritic cells (DCs) and macrophages, plays a major role in psoriasis establishment and progression [22].

Taking into account these mechanisms, psoriasis patients should be classified as suitable for topic or for systemic therapies (in case of moderate-to-severe disease): The latter group has to meet at least one criterion between more than 10% of the body surface area affected, psoriasis at special sites (scalp, face, palms and soles, or genitalia), and non-response to topical therapy [23]. Currently, available systemic therapies are usually small molecules interacting with ubiquitous intracellular targets, with a broad range of effects. For instance, methotrexate has been used for more than 50 years for the management of psoriasis and causes the inhibition of lymphocyte function by increasing adenosine production [24]. However, approved therapies for psoriasis are no longer limited to topical treatments or systemic therapies but are to date extended to the so-called “biologics”, which exert different action mechanisms on specific immune system components with a better efficacy rate [25]. The direct therapeutic targeting of TNF, IL-23, and IL-17 has proven to be clinically effective, albeit not without side effects and with an estimated rate of 50–70% of psoriatic patients who may show different response degrees to biologic drugs, reason why they are administered only in severe psoriasis cases and/or after developing psoriatic arthritis (occurring in 20/30% of cases after almost a decade from disease onset) [26,27].

Hence, modern oral anti-psoriatic drugs are still preferred for moderate psoriasis disease, as they can act as inhibitors interfering with intracellular proteins and affecting signaling pathways without direct targeting inflammatory mediators [25]. Dimethyl fumarate (DMF) represents the most important fumaric acid ester and the first modern compound clinically effective both in psoriasis and multiple sclerosis, two diseases in which T helper (Th) cell differentiation is promoted by IL-12 and IL-23 [28,29]. It has been demonstrated that its metabolite, monomethylfumarate (MMF), can activate nuclear factor kappa-light-chain-enhancer of activated B cells (NFkB) and prevents IL-12 and IL-23 production by macrophages and DCs, resulting in a decrease in neutrophils recruitment and keratinocytes proliferation, as well as causing the direct apoptosis of circulating T cells [25,30]. A consensus study [31] demonstrated that DMF treatment is characterized by dosage flexibility, sustained efficacy, high rates of drug survival, and low potential for drug–drug interactions, with the possibility of administration to a broad range of patients. However, the comprehension of the DMF mechanism of action needs to be integrated with the concept of a hijacked immune inflammatory microenvironment shaped by immune system dysregulation, which plays a pivotal role in psoriasis pathogenesis.

Hence, the aim of this study was to investigate the molecular mechanisms operated by immune populations located in the psoriatic plaque and to assess immune transcriptomic profile changes associated with DMF treatment (Skilarence^©^) in these lesions.

## 2. Materials and Methods

### 2.1. Case Selection

Nineteen patients diagnosed with moderate-to-severe psoriasis at the Dermatology Clinic and Pathology Unit of Città della Salute e della Scienza University Hospital of Turin between 2020 and 2021 were included in this study. Cases were selected by searching and evaluating their histological examinations on WinSAP 3.0 software (Engineering, Rome, Italy). Hematoxylin and eosin (H&E) slides from each case were reviewed by a pathologist confirming the histological type of psoriasis and grade of inflammation (defined as dermal infiltration of immune cells) by morphological characteristics. Clinicopathological data such as age at diagnosis, sex, familiarity, lesion site, number of involved sites, previous treatments, and follow-up data were collected from patients’ clinical reports in a dedicated and pseudonymized database.

### 2.2. RNA Extraction and Quantification

For each formalin-fixed paraffin-embedded (FFPE) tissue sample, a representative FFPE block was selected for subsequent analysis, and according to tissue dimensions, two to four 10 μm-thick sections were obtained from each block and collected in sterile Eppendorf tubes. RNA from 22 FFPE punch biopsies was extracted using RNAeasy FFPE Kit (QIAGEN Strasse 1, 40724 Hilden, Germany) according to product manufacturer instructions. The obtained RNA was quantified and assessed for purity (A260/280, A260/230) using a Nanodrop Spectrophotometer (Thermo Fisher Scientific, Waltham, MA, USA). 

### 2.3. Transcriptomic Analysis with NanoString Technologies

The transcriptomic profile was analyzed using the nCounter^®^ PanCancer Immune Profiling Panel (NanoString Technologies, Seattle, WA, USA) assessing 770 mRNA targets, including 40 housekeeping reference genes. This panel coverage comprises the main immune cell type genes, allowing comprehensive profiling of innate (macrophages, mast cells, neutrophils, NK cells, and the subset NK CD56dim cells) and adaptive (T cells, B cells, Th1 cells, Tregs, CD8+ T cells, exhausted CD8+ T cells, CD45+ cells, dendritic cells, and cytotoxic cells) immunity.

The analyses were set up according to the protocol provided by the manufacturer: for each sample, 50 to 300 µL of RNA were hybridized overnight at 70 °C with the NanoString Capture Probe and Reporter Probe (collectively called Codset). Samples were subsequently processed with the nCounter PrepStation, and the resulting cartridges were analyzed through the nCounter Digital Analyzer, a multi-channel epifluorescence scanner. Data were collected by taking images of the immobilized fluorescent reporters in the sample cartridge with a CCD camera through a microscope objective lens at the maximum data resolution, 550 fields of view (FOV).

Expression data were then normalized and analyzed with the nSolver Analysis Software (version 4.0, Nanostring Technologies, Siattle, WA, USA): For background correction, the mean count of negative controls plus two times the standard deviation was subtracted from the counts of each gene. The means of the supplied positive controls and the geometric mean of the housekeeping genes were used to normalize the measured expression values. Both positive and negative controls were included in the panel, according to the manufacturer’s instructions. Furthermore, the advanced analysis module (v. 2.0) was used to perform differential expression analyses using the nSolver Advanced Analysis module. Additional analyses were performed with GraphPad Prism software (v. 9.0, Prism, Boston, MA, USA) using the non-parametric Mann–Whitney test to compare the distribution of unmatched groups.

Out of 19, 5 patients were treated with DMF and they were analyzed with NanoString Technologies before (T0) and after 6 months (T6) of treatment to address immune-microenvironment changes. 

## 3. Results

### 3.1. Case Series

Out of 19 (57.9%), 11 patients were males and 8 (42.1%) were females, with a median age at diagnosis of 31.5 years old. The median PASI was 10. The subset of DMF-treated patients was composed of 3/5 (60%) males and 2/5 (40%) females, with a median age at diagnosis of 55 years old. Patients underwent DMF treatment at standard dosage for an average time of 10 months (minimum 8.8, maximum 14.6 months). Three patients suspended treatment due to lymphopenia after 10.1, 7.8, and 14.6 months, respectively. In Table 1, the main clinicopathological features are reported.

### 3.2. NanoString Molecular Analysis

**Figure 1:**
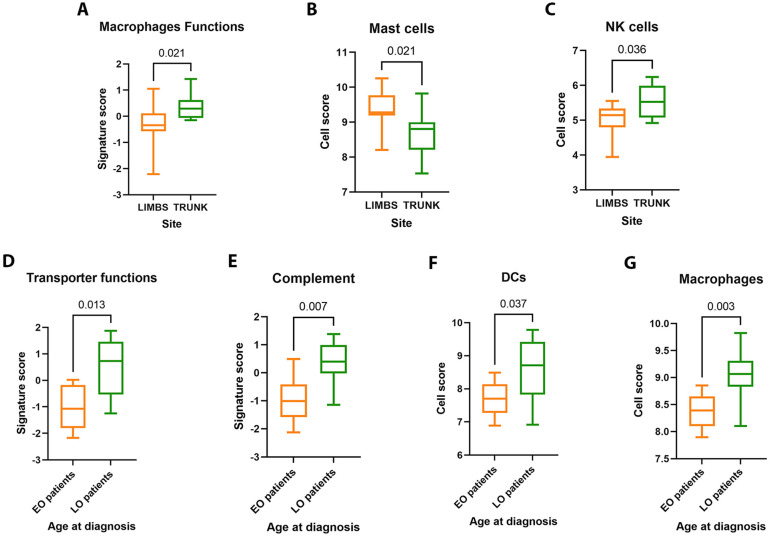
(**A**–**C**) Cell score and signature score analysis of limbs (*n* = 11) versus trunk (*n* = 6) biopsies. There is an abundance of expressed genes related to MCs, NK cells, and macrophage functions. (**D**–**G**) Comparison of EO (early onset, *n* = 6) versus LO (later onset, *n* = 13) patients: Cell score analysis reported a significant increase in macrophages and DCs activation in LO patients, while from signature score analysis emerged the activation of complement and transporter functions pathways.

We analyzed lesional skin biopsies comparing their molecular profile according to their site, PASI, and age onset. The first comparison between trunk biopsies and limb biopsies showed the upregulation of mast cells (MCs) and macrophage function signatures (*p* = 0.021) in trunk samples, while natural killer (NK) cells were enriched in limb biopsies (*p* = 0.031) (Figure 1A). Neutrophils and MCs represent the main sources of IL-17 in psoriatic lesions [32]. At the early stage of the inflammatory process, MCs can produce cytokines to activate keratinocytes and are responsible for neutrophil chemotaxis into the epidermis [33]. They also activate DC and stimulate CD4+ T cells to release interferon-gamma (IFN-γ) and IL-17, hence contributing to psoriasis pathogenesis. In addition, keratinocytes secrete chemokines responsible for the recruitment of NK cells, which in turn sustain inflammatory response through the production of IL-12, IL-15, IL-18, and IL-23 [34]. Thus, the evidence of a different site-related distribution of innate immune cells, respectively involved in the initiation of psoriasis and establishment of a cytotoxic environment, might suggest a link with more or less advanced stages of the disease.

After stratifying patients according to the PASI cut-off of 10, no significant results were observed, meaning no link between molecular expression of immune profile and clinical amount of skin disease. However, when comparing early-onset (EO) with later-onset (LO) patients with a cut-off based on median age onset of 32 years old, a significantly higher presence of macrophages (*p* = 0.032) and DC cells (*p* = 0.037) was detected in LO patients, as well as an increase in complement (*p* = 0.07) and transporter function (*p* = 0.025) pathways (Figure 1B). Macrophages are involved in the initial phase of psoriasis development and promote the maturation and activation of myeloid DCs, which play an important role in chronic disease [35]. On the other hand, the complement factor C3a is significantly expressed in patients with psoriasis, suggesting a continuous activation of the complement system resulting in the production of inflammatory mediators [36]. Even if there is no data regarding molecular mechanisms underlying age onset in psoriasis, a correlation between genetic predisposition (i.e., positive family history) and clinical presentation has been demonstrated [37]. Our results suggest that EO and LO patients display a different inflammatory immune microenvironment which may affect psoriasis clinical course.

### 3.3. Molecular Profile of DMF-Treated Patients

DMF activity has been evaluated through both clinical and pathological parameters. The treatment successfully reduced the PASI and tissue inflammation. Indeed, the median PASI for T0 and T6 biopsies was 10 and 0, respectively.

**Figure 2:**
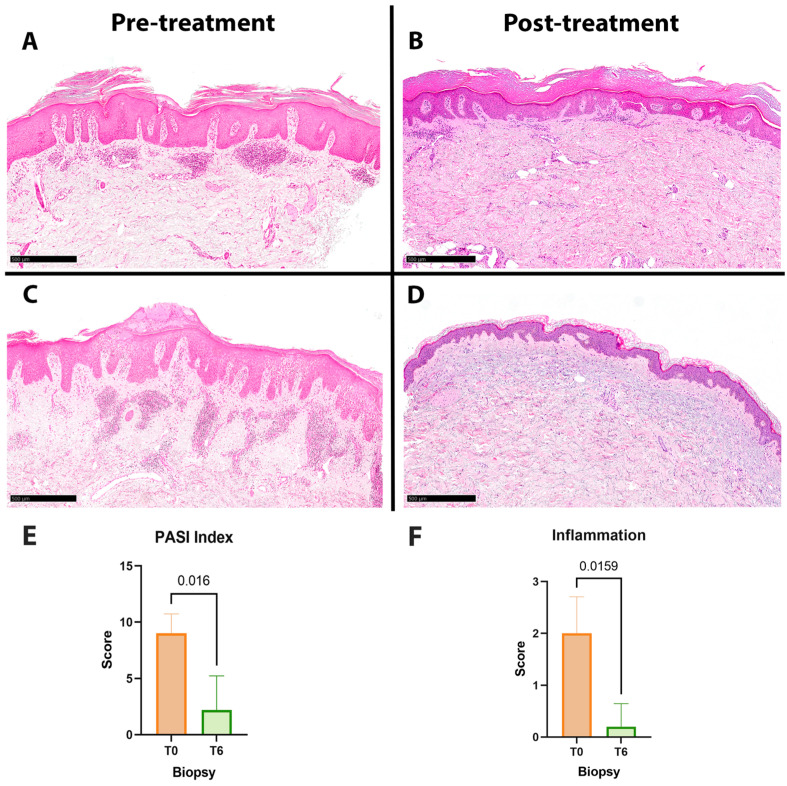
Skin biopsies before ((**A**,**C**) H&E 50×, scale bar: 500 μm) and after ((**B**,**D**) H&E 50×, scale bar: 500 μm) DMF treatment of two representative samples: In both reported cases, an evident decrease in inflammation occurred in post-treatment specimens. The average distribution of PASI (**E**) and inflammation score (**F**) before (T0 biopsies, *n* = 5) and after (T6 biopsies, *n* = 5) 6 months of DMF treatment showed a notable change which reflected the histological aspect.

In histology, DMF activity was assessed based on the degree of inflammation using a semiquantitative score ranging from 0/1+ (absent/mild inflammation) to 2+/3+ (moderate/intense inflammation) (Figure 2A–D). The median PASI decreased to 0 after treatment (*p* = 0.016), as well as the median inflammation score from 2 to 0 in T6 biopsies (*p* = 0.016) (Figure 2E,F).

**Figure 3:**
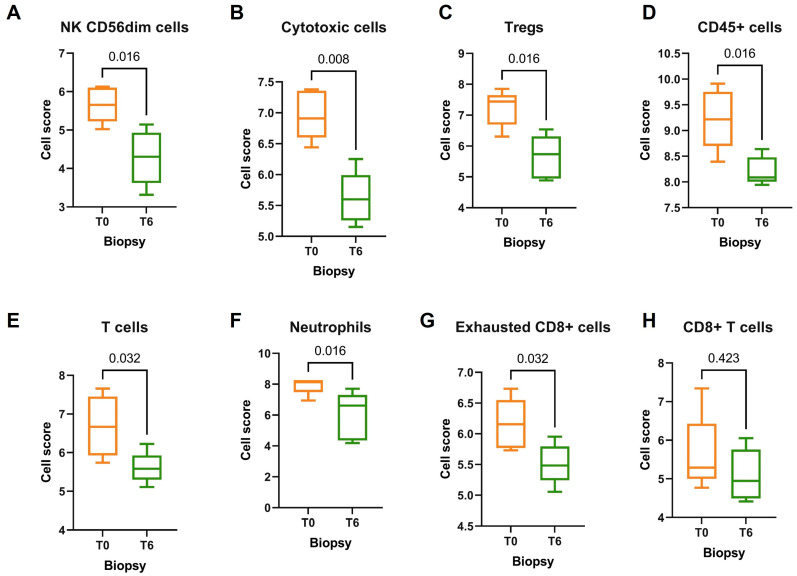
(**A**–**H**) Cell score analysis of T0 (*n* = 5) versus T6 (*n* = 5) biopsies.

**Figure 4:**
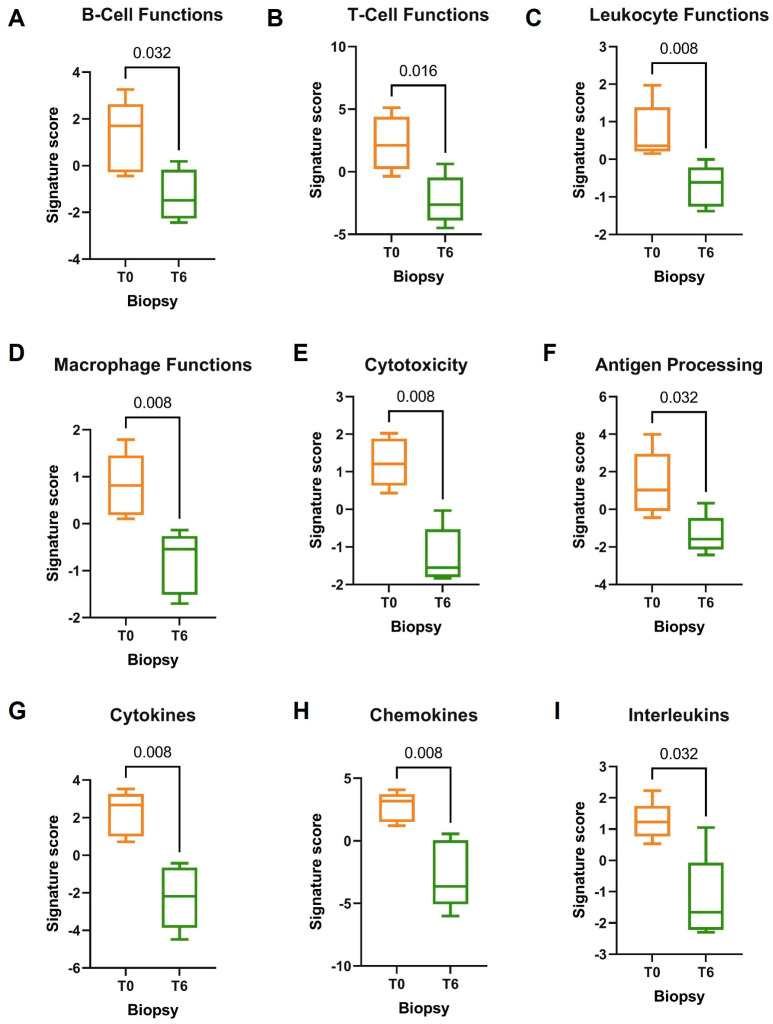
(**A**–**I**) Pathway score analysis of T0 (*n* = 5) versus T6 (*n* = 5) biopsies.

The comparison between 5 T0 biopsies and 5 T6 biopsies demonstrated a strong immunomodulatory effect of DMF on inflammatory cells and pathways. Several signatures correlated to immune cell types were highly expressed in T0 biopsies rather than in T6 biopsies: Despite the higher levels of T cells (*p* = 0.032) and cytotoxic cells (*p* = 0.079) detected in T0 biopsies, we did not observe any differences in the subgroup of CD8+ T cells distribution (*p* = 0.421). However, there was an abundance of exhausted CD8+ T cells in T0 biopsies (*p* = 0.032), probably reflecting the inflammatory process that psoriatic plaque underwent before treatment, also sustained by the enrichment of NK CD56dim cells, Tregs, neutrophils, and CD45+ cells (*p* = 0.016) (Figure 3). Regrettably, no significant differential gene expression was observed in the general comparison between T0 and T6 groups due to the limited cohort. However, there was a significant decrease in numerous signaling pathways in T6 biopsies, mainly involved in the regulation of T cells (*p* = 0.016), B cells (*p* = 0.032), macrophages, and leucocytes functions (*p* = 0.0079) (Figure 4). T6 biopsies were also characterized by a strong immunoregulatory activity reflected by the reduction in several pro-inflammatory pathways, such as cytotoxicity (*p* = 0.008), pathogen defense (*p* = 0.008), antigen processing (*p* = 0.032), adhesion (*p* = 0.008), and cell cycle (*p* = 0.016), as well as key pro-inflammatory markers like chemokines (*p* = 0.008), cytokines (*p* = 0.008), and interleukins (*p* = 0.032) (Figure 4).

## 4. Discussion

Psoriasis is a chronic disease caused by the dysregulation of immune system homeostasis. Nowadays, the understanding of its complex architecture increased the number of effective therapeutic options available to patients. Traditional therapies, including methotrexate and cyclosporin, induce a general immunosuppression by preventing T-cell activation, but long-term use is often associated with several toxicities [25]. Fumaric acid esters (FAEs) like DMF, instead, act on the immune system by inhibiting the production of pro-inflammatory cytokines, such as IL-12, IL-17, and IL-23, achieving better results in terms of clinical efficacy and tolerance [29,38]. These encouraging data open the opportunity for the next generation of oral compounds to enter daily clinical practice in psoriasis, extending the list of therapeutic options for those patients. However, the immune and molecular mechanism influenced by these compounds still needs to be further investigated, from the perspective of assigning the appropriate treatment according to patients’ needs and characteristics.

Our work aimed to uncover possible correlations between some clinical features and the immune microenvironment of psoriatic lesions to address the lack of knowledge about their molecular landscape. Even though the main clinical parameter exploited for severity assessment (PASI) did not correlate with biological data, molecular profile analysis of our case series differed by age onset and psoriasis site involvement, suggesting that psoriasis clinical course may be affected by a different composition in immune cell populations and activated inflammatory signaling of psoriatic plaques. Moreover, the subset of patients treated with DMF showed a promising reduction in inflammatory microenvironment due to impaired induction of both immune cells and their corresponding pathways. Treumer et al. showed that DMF exerts its anti-inflammatory action on psoriatic plaques by inducing T-cell apoptosis through the modulation of NFκB signaling [39]. Our data confirmed DMF inhibitory action on leucocyte function and cytotoxicity but also demonstrated its influence on the expression of circulating molecules (e.g., cytokines, chemokines) and distinct inflammation pathways. In particular, we observed a DMF-mediated reduction in antigen processing and presentation signaling. This may also suggest an indirect inhibition of T cells through the modulation of the inflammatory microenvironment towards immune tolerance and a less activated functional state. However, the reduction in Tregs after treatment may be controversial, according to the literature. The link between the number of Tregs and psoriatic disease is currently debated: Although the impairment of Tregs suppressive function is known to lead to an altered Th17/Treg balance in psoriasis, several publications reported variable results. Zhang et al. observed a correlation between the increase in Tregs in psoriatic skin with PASI scores [40], while Bovenschen et al. described a higher frequency of CD4+ CD25+ Foxp3+ Tregs in the dermis of patients with plaque psoriasis [41]. In contrast, Yun et al. found a decrease in Foxp3+ Tregs in lesional skin of patients with acute disease, but an increase in patients with chronic disease [42], while Yan et al. reported that Foxp3+ Tregs were increased in plaque psoriasis but decreased in the guttate subtype when compared with normal skin [43].

These conflicting results may reflect different disease states, sites of biopsy, psoriasis subtypes, and skin tissue sampling. Moreover, it is important to note that our analyses were carried out using NanoString Technologies, a bulk method that allows assessing the transcriptomic expression of a tissue in its entirety. The heterogeneous composition of skin lesional biopsies may have played a role in determining the percentage of observed cell genes, and to further investigate Tregs composition, it would be necessary to choose a single-cell resolution technique, which may provide novel insights on this debated theme.

On the other hand, the main downside of this treatment remains lymphopenia, which was developed by 3 out of 5 patients. The underlying mechanism remains to be elucidated, although the interaction between DMF metabolite, monomethylfumarate (MMF), and the receptor HCA2 expressed both by myeloid and tissue-resident cells may play a role [30]. Nevertheless, several works investigated the possible link between lymphocyte count and the success of DMF treatment, demonstrating that a reduced number of lymphocytes is associated with better clinical efficacy of this therapy, in line with our results [44,45,46,47].

## 5. Conclusions

In conclusion, our preliminary data overall show that a different tumor immune microenvironment is associated with age onset and plaque site, and this may influence the clinical course of psoriasis without necessarily being reflected in exclusively clinical parameters like the PASI. Moreover, DMF allows the achievement of an immune-tolerant and non-inflammatory condition through the modulation of both innate and adaptive immunity, representing a valuable clinical option for psoriasis patients.

## Figures and Tables

**Table 1:** Clinical features of DMF-treated patients.

	Age	Sex	Involved Sites	T0 PASI	T6 PASI	T0 Inflammation Score	T6 Inflammation Score
Patient 1	40	F	Face, scalp	6	0	1	0
Patient 2	75	M	Face, buttock, scalp, lower limbs	10	0	2	0
Patient 3	56	M	Genitals, scalp, upper limbs, lower limbs	10	5	2	1
Patient 4	43	F	Trunk, buttock, upper limbs	10	0	3	0
Patient 5	60	M	Back, plantar	9	6	2	0

## Data Availability

The collected data are not publicly available to protect patients’ privacy and comply with ethical requirements. Aggregated data supporting the study findings are available from the corresponding author upon a reasonable request.
